# S184. MACHINE LEARNING REVEALS DEVIANCE IN NEUROANATOMICAL MATURITY PREDICTIVE OF FUTURE PSYCHOSIS IN YOUTH AT CLINICAL HIGH RISK

**DOI:** 10.1093/schbul/sby018.971

**Published:** 2018-04-01

**Authors:** Yoonho Chung, Jean Addington, Carrie Bearden, Kristen Cadenhead, Barbara Cornblatt, Daniel Mathalon, Thomas McGlashan, Diana Perkins, Larry Seidman, Ming Tsuang, Elaine Walker, Scott Woods, Sarah McEwen, Theo van Erp, Tyrone Cannon

**Affiliations:** 1 Yale University; 2 University of Calgary; 3 University of California, Los Angeles; 4 University of California, San Diego; 5 The Zucker Hillside Hospital; 6 University of California, San Francisco; 7 University of North Carolina; 8 Harvard Medical School; 9 Emory University; 10 University of California, Irvine

## Abstract

**Background:**

Both early (pre- and perinatal) and late (adolescent) neurodevelopmental disturbances are hypothesized to contribute to the pathophysiology of schizophrenia. Disturbances originating earlier in life (e.g., resulting from the interplay of genetic factors and obstetric complications) would be expected to affect brain integrity from birth onwards and could therefore help to explain cases with subtle deficits in premorbid functioning during childhood and earlier ages at onset of full psychosis (i.e., early to mid-teens). In contrast, disturbances that emerge during late adolescence and early adulthood (e.g., via abnormal neuromaturational events and/or environmental factors) could help to explain cases with normal premorbid psychological health and a more acute onset of psychotic symptoms and functional impairment in the late teens and early twenties. However, it is yet unclear whether neuroanatomical data among individuals at clinical high risk (CHR) for psychosis can be modeled to detect early versus late neurodevelopmental influences that is predictive of future psychosis onset. Therefore, in this study, we investigated whether the timing of the appearance or course of the deviation from normal brain maturation, as determined using a machine learning algorithm trained on structural MRI data to estimate age, is potentially relevant to the early versus late neurodevelopmental framework among CHR individuals.

**Methods:**

A neuroanatomical-based age prediction model was trained using a supervised machine learning technique with T1 MRI scans from 953 typically developing healthy controls (HC) from the Pediatric Imaging, Neurocognition, and Genetics study (PING) study. The trained model was then applied to 109 HCs and 275 CHR, including 39 converters (CHR-C), from the North American Prodrome Longitudinal Study (NAPLS2) and 14 cases of first episode psychosis patients (FE) for external validation and clinical application. Discrepancy between neuroanatomical-based estimated age and chronological age was computed for each individual (i.e., brain age gap) and compared across clinical groups.

**Results:**

The PING-derived model for estimating age accurately predicted NAPLS HC subjects’ chronological ages, explaining 51% of the variance (P < 0.001) in chronological age, with a mean absolute error of 1.41 years, providing evidence of independent external validation. CHR subjects and FE adolescents showed a significantly greater overestimated gap between model-predicted age and chronological age compared with HC (Ps < 0.01). This effect was significantly moderated by chronological age, with neuroanatomical-based estimated age systematically overestimating CHR cases aged 12–17 years, but not among those aged 18–21 years. In the ROC analysis, brain age gap was a significant predictor of conversion to psychosis with an area under the curve of 0.63 (P < 0.05) among younger adolescents. In addition, increased deviation of brain age gap predicted pattern of stably low functioning over time (P < 0.05) among CHR individuals. In contrast, previously reported evidence of an accelerated reduction in cortical thickness among CHR-C was found to apply only to those cases who were 18 years or older.

**Discussion:**

These results are consistent with the view that both early and later neurodevelopmental disturbances contribute to the onset and course of schizophrenia, with the two sets of influences having differing implications for the intercepts and trajectories in structural brain parameters as a function of age. The results also suggest that baseline neuroanatomical measures are likely to be useful in prediction of psychosis especially (or only) among CHR cases who are below 18 years of age at the time of ascertainment.

